# Differentiating Benign from Malignant Causes of Splenomegaly: Is Acoustic Radiation Force Impulse Elastography Helpful?

**DOI:** 10.3390/diseases12120308

**Published:** 2024-11-30

**Authors:** Amjad Alhyari, Oussama Dob, Ehsan Safai Zadeh, Christoph Frank Dietrich, Corrina Trenker, Thomas M. Gress, Christian Görg

**Affiliations:** 1 Gastroenterology, Endocrinology, Metabolism and Clinical Infectiology, University Hospital Giessen and Marburg, Philipp University of Marburg, Baldingerstraße, 35037 Marburg, Germany; thomas.gress@med.uni-marburg.de (T.M.G.); christian.goerg@uk-gm.de (C.G.); 2 Interdisciplinary Centre of Ultrasound Diagnostics, University Hospital Giessen and Marburg, Philipp University of Marburg, Baldingerstraße, 35037 Marburg, Germany; trenker@med.uni-marburg.de; 3 Department of Radiology, University Hospital Giessen and Marburg, Philipp University of Marburg, 35037 Marburg, Germany; oussama.dob@uk-gm.de; 4 Department of Biomedical Imaging and Image-Guided Therapy, Medical University of Vienna, 1019 Vienna, Austria; ehsan.safaizadeh@meduniwien.ac.at; 5 Department Allgemeine Innere Medizin (DAIM), Kliniken Hirslanden Bern, Beau Site, Salem und Permanence, 3013 Bern, Switzerland; c.f.dietrich@googlemail.com; 6 Hematology, Oncology and Immunology, University Hospital Giessen and Marburg, Philipps University Marburg, Baldingerstraße, 35037 Marburg, Germany

**Keywords:** ARFI, spleen, point shear wave elastography, ultrasound, splenomegaly

## Abstract

Purpose: To evaluate the ability of acoustic radiation force impulse (ARFI) elastography in differentiating benign from malignant etiologies of splenomegaly based on differences in splenic stiffness. Materials and Methods: Between September 2020 and November 2022, we evaluated 40 patients with splenomegaly—defined by a splenic long axis greater than 13 cm and/or a short axis greater than 6 cm, without visible focal or infiltrative mass lesions—using abdominal ultrasound at our university hospital. Each patient also underwent a standardized ARFI elastographic assessment of the enlarged spleen, with data collected prospectively. We then retrospectively analyzed the cases with confirmed etiologies of splenomegaly from their final medical reports. Mean ARFI velocities (MAV) were compared across patients with splenomegaly due to malignant infiltration (MIS) from hematological malignancy, congestive splenomegaly (CS) due to portal or splenic vein congestion/occlusion, and immune-related splenomegaly (IRS) associated with systemic infectious or autoimmune diseases. Results: Among the 40 patients with splenomegaly, 21 (52.5%) were diagnosed with malignant infiltrative splenomegaly (MIS), 11 (27.5%) with congestive splenomegaly (CS), and 8 (20%) with immune-related splenomegaly (IRS). The mean ARFI velocities (MAV) for the MIS, CS, and IRS groups were 3.25 ± 0.68 m/s, 3.52 ± 0.47 m/s, and 2.84 ± 0.92 m/s, respectively. No significant differences were observed in splenic stiffness (MAV) among these groups. Conclusions: Differentiating between benign and malignant etiologies of splenomegaly based on stiffness differences observed in ARFI elastography is not feasible. Larger prospective studies are necessary to validate these findings.

## 1. Introduction

The spleen is a vital organ involved in immune function and blood filtration. Histologically, it is characterized by two main areas: the red pulp and white pulp. The red pulp is responsible for filtering blood, removing old or damaged red blood cells, and recycling iron. It consists of a network of sinusoids and splenic cords. The white pulp, on the other hand, is part of the immune system, containing lymphocytes and playing a role in producing antibodies. Surrounding these structures is a fibrous capsule, with trabeculae extending into the organ to provide support. The white pulp surrounds the central arterioles and is composed of lymphoid follicles, where immune responses are initiated. Marginal zones between the red and white pulp allow for the interaction between blood and immune cells. This balanced structure enables the spleen to perform its dual role in both immune surveillance and blood filtration, making the spleen susceptible to a variety of systemic diseases [1,2].

Splenomegaly has a prevalence of about 2% in adults [3] and can result from a range of benign and malignant conditions, each with distinct pathophysiological mechanisms. These include the following: 1. Splenic venous congestion due to liver diseases, such as cirrhosis and hepatitis, or other causes of portal hypertension, including portal or splenic vein thrombosis and congestive heart failure. 2. Splenic sequestration of abnormal blood cells, seen in conditions like sickle cell disease, hemolytic anemia, and thalassemia. 3. Benign infiltrative causes such as sarcoidosis and storage diseases (e.g., Gaucher disease and Niemann–Pick disease). 4. Immune-mediated causes from infections (acute or chronic viral, bacterial, or parasitic infections) or autoimmune disorders, including systemic lupus erythematosus, rheumatoid arthritis, and other cytopenia-related conditions. 5. Malignant infiltrative causes, including lymphomas, leukemias, amyloidosis, and myeloproliferative disorders. 6. Solid or cystic focal lesions of the spleen, which may be metastatic or primary, infectious, or congenital in origin [3,4,5,6,7].

Given the extensive differential diagnosis for splenomegaly and the often absent or nonspecific symptoms, the diagnostic workup can be challenging and time-consuming [4]. Moreover, while non-splenic pathologies can be further evaluated through tissue biopsies, obtaining splenic biopsy is not a straightforward decision due to the well-documented high risk of bleeding [8,9]. Initial testing should include testing for Epstein–Barr virus, a complete blood count, a comprehensive metabolic panel, and abdominal ultrasound [4]. Further testing may include extensive hematological, immunological, biochemical, and radiological assessments. Regular follow up, splenic biopsy or even splenectomy may sometimes be required [4,10].

Imaging plays a crucial role in the evaluation of splenomegaly. Techniques such as B-mode ultrasound (B-US), color Doppler ultrasound, and contrast-enhanced ultrasound (CEUS) are cost-effective bedside options for assessing spleen size, vascularity, the tissue composition of lesions, and the extent of splenic involvement (focal, partial, or global). These methods can also offer insights into the nature of splenic involvement, whether benign or malignant, and help identify any hepatoportal causes [4,10]. Computed tomography (CT) with contrast, magnetic resonance imaging (MRI), and positron emission tomography (PET) are also invaluable imaging tools in evaluating the presence of focal lesions and extra-splenic systemic involvement (e.g., lymphadenopathy) [10].

Guidelines have been established for using spleen elastography in patients with liver disease, particularly for evaluating clinically significant portal hypertension and esophageal varices [11,12,13]. However, there are little conflicting data on the value of elastography in predicting the etiology of splenomegaly [14,15,16,17]. Webb et al. reported no significant differences between splenomegaly patients with benign and those with malignant etiologies [15]. On the other hand, Batur et al. and Yalçın et al. reported significant differences in median splenic stiffness between splenomegaly due to benign versus malignant etiologies [14,16].

In this study, we intended to evaluate the ability of US elastography using acoustic radiation force impulse (ARFI) as a non-invasive quantitative imaging modality in differentiating between benign and malignant etiologies of splenomegaly based on differences in splenic tissue stiffness.

## 2. Patients and Methods

### 2.1. Study Design

This standardized study, conducted between September 2020 and November 2022, involved a retrospective data analysis of 40 consecutive patients with splenomegaly (defined as a spleen length ≥ 13.0 cm and/or spleen thickness ≥ 6 cm) who underwent abdominal ultrasound at the ultrasound department of the University Hospital of Marburg. Elastographic examinations were performed to assess the tissue stiffness of the enlarged spleens. The study was approved by the local ethics committee at Philipps University of Marburg (protocol code: 24-256 RS) and complied with the amended Declaration of Helsinki. Informed consent was obtained from all patients for both ultrasound and elastographic examinations.

### 2.2. B-US Examinations

All examinations, including B-US and ARFI elastography, were conducted using the Siemens Acuson S2000 and Acuson S3000 systems (Siemens Medical Solutions, Erlangen, Germany) by two independent, qualified investigators (E.S., A.A.) under the supervision of a Level III-qualified examiner from the German Society for Ultrasound in Medicine (DEGUM), C.G., who is an expert in internal medicine with over 39 years of sonographic experience. With the patient fasting, a left lateral intercostal approach of the transducer was used while the patient lay in a supine position. A curvilinear 6C1 transducer was applied to visualize the spleen. Once an optimal visualization of the enlarged spleen was achieved, adjustments to focus and gain were made as needed. The spleen’s maximum length and thickness were then measured in centimeters and documented.

### 2.3. ARFI Examinations

**Figure 1:**
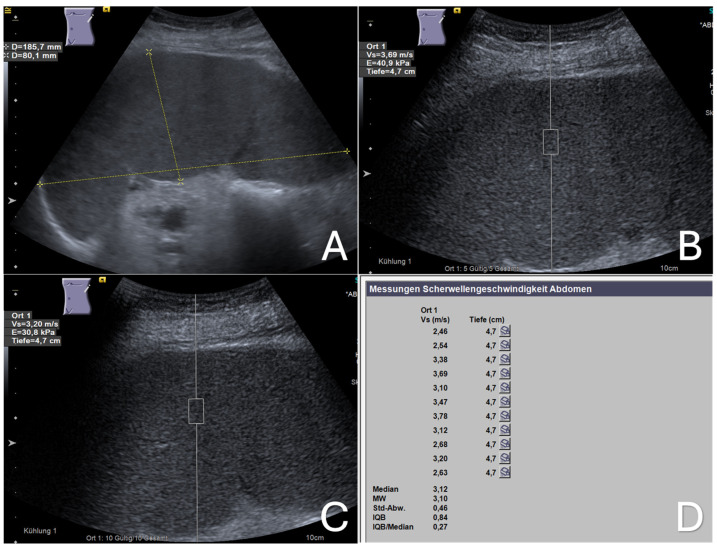
Benign congestive splenomegaly. A 52-year-old male patient with liver cirrhosis-related portal hypertension. (**A**) B-mode ultrasound image of the enlarged spleen with a length of 18.6 cm and a thickness of 8.0 cm; (**B**,**C**) two elastographic images of enlarged spleen showing shear wave velocities (Vs) of 3.69 and 3.20 m/s, respectively; (**D**) the final elastographic report of the enlarged spleen, showing a mean shear wave velocity (MW) of 3.10 m/s. Ort 1: location 1; Vs (m/s): velocity in meter per second; Tiefe (cm): depth in centimeter; MW: mean value (Mittelwert); Std-Abw.: standard deviation (Standard Abweichung); IQB: interquartile range (Interquartilbereich).

**Figure 2:**
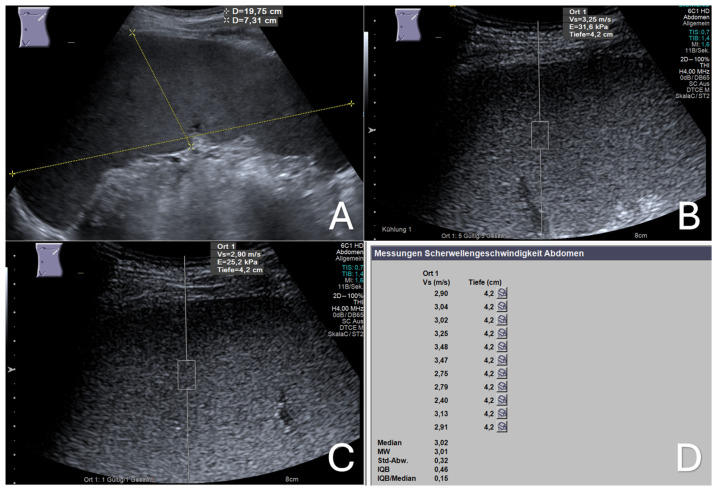
Malignant infiltrative splenomegaly. A 53-year-old male patient with myeloproliferative neoplasm (polycythemia vera). (**A**) B-mode ultrasound image of the enlarged spleen with a length of 19.8 cm and a thickness of 7.3 cm; (**B**,**C**) two elastographic images of enlarged spleen showing shear wave velocities (Vs) of 3.25 and 2.90 m/s, respectively; (**D**) the final elastographic report of the enlarged spleen, showing a mean shear wave velocity (MW) of 3.01 m/s. Ort 1: location 1; Vs (m/s): velocity in meter per second; Tiefe (cm): depth in centimeter; MW: mean value (Mittelwert); Std-Abw.: standard deviation (Standard Abweichung); IQB: interquartile range (Interquartilbereich).

With the patient fasting, a left lateral intercostal approach of the transducer was used while the patient lay in a supine position. After the optimal ultrasound visualization of the enlarged spleen, the depth settings were modified, bringing the central area of the spleen without visible vessels to the center of the ultrasound screen; the region of interest (ROI), represented by a box measuring 10 × 5 mm, was then positioned within this area, at least 2 cm under the splenic capsule. For each measurement acquisition, the patient was instructed to hold his/her breath during mid-expiration for at least six seconds. The measurement was displayed as velocity in meters per second (m/s) on the upper corner of the screen. If the patient moved or breathed during measurement acquisition, that reading was deemed invalid, and a new measurement was taken. A total of 11 measurement acquisitions were obtained from different points of the displayed splenic area on the screen, and the mean ARFI velocity (MAV) of the 11 measurement acquisitions was calculated and registered, as shown in Figure 1 and Figure 2.

### 2.4. Final Diagnosis of Etiology of Splenomegaly

The etiologies of splenomegaly were classified into three main groups: 1. malignant infiltrative splenomegaly (MIS) due to hematological malignancy; 2. congestive splenomegaly (CS) due to portal or splenic vein congestion or thrombosis; and 3. immune-related splenomegaly (IRS) due to systemic infectious or autoimmune disease.

The diagnostic confirmation of the etiology of splenomegaly was based on the microscopic examination of peripheral blood and bone marrow samples in the MIS group, clinical and imaging findings of cirrhosis and/or radiologic features of splenic/portal venous dilatation or thrombosis in the CS group, and clinical, microbiological, and serological tests revealing the infectious cause or the presence of an autoimmune disease in the IRS group. Reference imaging (CT and/or MRI) was available in 36/40 (90.0%) of all patients.

**Figure 3:**
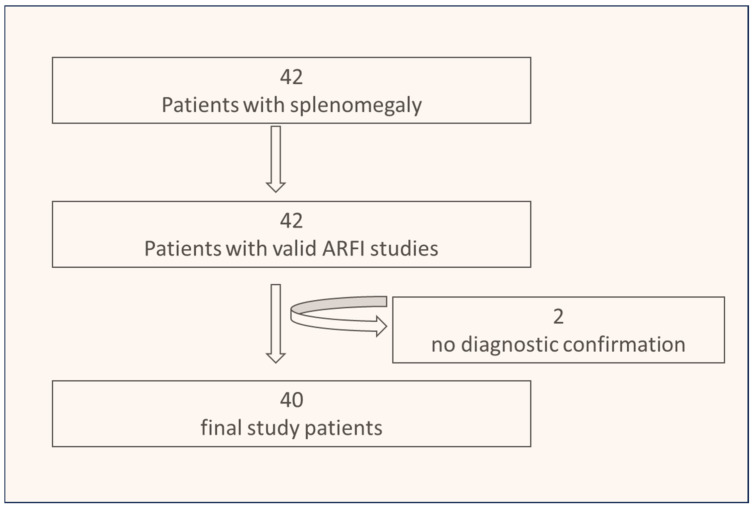
Flow diagram of the study patients.

In total, 2/42 patients (4.8%) were excluded due to the lack of definitive diagnostic confirmation; see Figure 3. Baseline clinical and sonographic characteristics as well as final diagnoses in all patients are depicted in Table 1. 

### 2.5. Statistical Analysis

All statistical analyses were performed using Excel (Microsoft 365 MSO) and SPSS version 26.0. Numerical data are presented as mean ± standard deviation (SD). Categorical variables were analyzed using Fisher’s exact test, while continuous variables were assessed using the Mann–Whitney test. Spearman’s rank correlation and multiple regression analysis were employed to evaluate significant correlations between continuous variables. A *p*-value of less than 0.05 was considered statistically significant.

## 3. Results

### 3.1. Demographics

Of the 40 study patients, 25 (62.5%) were males and 15 (37.5%) females. The mean age was 53 ± 17 years (range 24–78 years). The mean body mass index (BMI) was 26.7 ± 4.3 Kg/m^2^ (range: 13.9–36.7 Kg/m^2^). The cause of splenomegaly was more likely to be malignant in older subjects (*p* = 0.002). On the other hand, no significant associations were found between sex or BMI and frequency of malignancy (*p* > 0.05).

### 3.2. B-US

While no significant differences in splenic length were found between benign (15.6 ± 2.1 cm) and malignant (18.5 ± 5.8 cm) causes of splenomegaly (*p* = 0.16). The spleen thickness was larger in those of malignant splenomegaly (6.6 ± 1.4 vs. 9.1 ± 2.6 cm; *p* = 0.001).

### 3.3. ARFI

**Figure 4:**
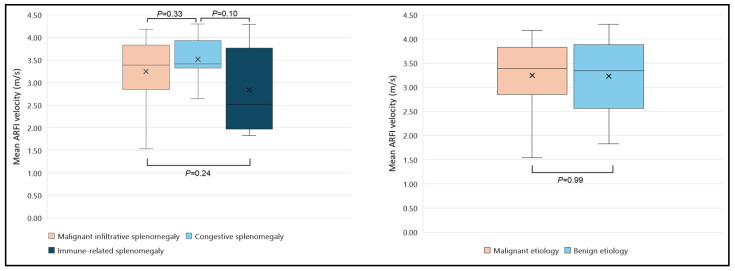
Comparison of mean ARFI velocities of spleen between benign and malignant groups (**right**) different subgroups (**left**). The mean is represented by an “X” in each box, and the median in different groups is represented by the horizontal line within each box. ARFI, acoustic radiation force impulse.

First, Spearman’s rank correlation and multiple regression analysis were used to examine the correlations between age, splenic size, and spleen elasticity, as measured by mean ARFI velocities (MAV). Both tests did not show a significant influence of the patient’s age or the length of the long or short splenic axes on MAV (*p* > 0.05). MAV were 3.25 ± 0.68 m/s in the MIS group, 3.52 ± 0.47 m/s in the CS group, and 2.84 ± 0.92 m/s in the IRS group. No significant differences in MAV were found between the benign (*n* = 19) and malignant (*n* = 21) etiologies (3.23 ± 0.76 vs. 3.25 ± 0.68 m/s, respectively, *p* = 0.99). Likewise, MAV did not differ between the MIS and the CS groups (*p* = 0.33), the MIS and the IRS groups (*p* = 0.24), or the IRS and the CS groups (*p* = 0.1). A summary of the elastographic results among different patients’ groups is shown in Figure 4 and Table 2.

## 4. Discussion

The spleen plays a vital role in filtering blood, recycling red blood cells, and mounting immune responses, making it susceptible to a variety of systemic diseases [2]. Splenomegaly can result from a wide range of conditions and the differentiation between benign and malignant causes of a splenic enlargement is crucial for accurate treatment and better patient outcomes [3,10]. However, this differentiation can be challenging due to overlapping clinical and imaging findings, and a thorough analysis of clinical features, laboratory tests, and imaging techniques is needed to make the diagnosis, and sometimes splenic biopsy, or even splenectomy, is necessary [8].

Apart from estimating the size of the spleen in the detection and follow up of splenomegaly, detection, and characterization of focal splenic lesions as well as the presence of related extra-splenic pathologies (e.g., lymphadenopathy, hepatic, or portal diseases), the role of imaging in establishing the etiology of splenomegaly is limited [4].

Ultrasound is a cost-effective and radiation-free method which is the first line and the most reliable imaging technique in determining the size of the spleen [4,10]. In the current study, no significant differences in the length of the spleen between benign and malignant etiologies were found. Interestingly, however, there were significant differences in thickness in which the splenomegaly of those with underlying malignant etiology demonstrated greater splenic thickness than those with benign underlying etiology.

Ultrasound elastography is a promising non-invasive technique for evaluating tissue stiffness and has shown significant utility in a variety of organs, including the liver, thyroid, pancreas, kidneys, breast, lymph nodes, and prostate [11,18]. In previous research, we demonstrated the feasibility and diagnostic value of Acoustic Radiation Force Impulse (ARFI) elastography in distinguishing benign from malignant lesions in the mesentery [19], omentum [20], and peripheral lung lesions [21].

In a previous study, we also explored the feasibility and diagnostic performance of ARFI elastography in characterizing focal splenic lesions and found no significant differences in stiffness values between benign and malignant lesions [22].

As mentioned earlier, there are limited and conflicting data on the role of elastography in predicting the etiology of splenomegaly [14,15,16,17]. In the current study, consistent with the findings of Webb et al., we were unable to identify significant differences in mean stiffness values (MAV) between benign and malignant causes of splenomegaly. Similarly, no significant differences in MAV values were observed across the different categories (CS, IRS, and MIS). Table 3 summarizes previous studies on the use of US elastography in etiological characterization in patients with splenomegaly.

Splenic stiffness in healthy volunteers was reported to be significantly lower than that in patients with CS and MIS using transient, 2D, and ARFI elastographies [14,15,16,23]. We did not include healthy individuals in our study and we aimed to find differences in patients with pathological splenomegaly.

Splenic stiffness was shown to be highly variable in different subjects. For instance, the reported values for normal splenic stiffness in healthy volunteers vary widely across different studies [24,25]. Variation in sinusoidal congestion and/or perisinusoidal fibrotic changes in the spleen may be a contributing factor to these observations [26,27]. Additionally, parenchymal stiffness in patients with CS is variable depending on the degree of portal hypertension and the effectiveness of treatment like TIPS [13] and non-selective beta blockers [28]. Similarly, the splenic stiffness of MIS patients varies according to disease burden [23,24,29,30] and treatment stage [29]. The patients who were retrospectively included in the current study were located at different stages of treatment for portal hypertension in the CS group and chemotherapy in the MIS group, which may have probably affected the splenic stiffness value.

Nevertheless, ultrasound elastography in patients with splenomegaly can still be implemented to detect changes in splenic stiffness over time, which can be helpful in monitoring disease progression or the response to treatment in both patients with hepatoportal-related splenomegaly [11,31], as well as patients with myelofibrotic- and hematological-related splenomegaly [24,29,30,31].

This study has several limitations: There was no analysis of interobserver variability. However, previous studies have demonstrated the reproducibility of ARFI measurements in various organs [32,33]. Additionally, due to the retrospective design, the examiners were not blinded to the clinical and radiological data, potentially introducing bias. Moreover, this was a single-center study from a tertiary university hospital with a relatively small patient sample size, which may limit its generalizability. Therefore, further large-scale prospective studies, preferably comparing ARFI with other cross-sectional imaging modalities, are necessary to validate these findings.

## 5. Conclusions

Although the ARFI elastography of the spleen has recently shown a promising potential in staging hepatoportal- and myeloproliferative-related splenomegaly and monitoring their response to therapy, it was unable to differentiate between the benign and malignant causes of splenomegaly. More studies on the elastography of the spleen are needed to confirm our findings.

## Figures and Tables

**Table 1:** Baseline clinical and sonographic characteristics and final diagnoses in 40 study patients.

Group.	Benign Causes (*n* = 19)	Malignant Causes (*n* = 21)
Mean age (years)	45 ± 16	61 ± 15
Mean BMI (kg/m^2^)	26.7 ± 3.8	26.7 ± 4.9
Male/Female	13/6	12/9
Mean splenic length (cm)	15.6 ± 2.1	18.5 ± 5.8
Mean splenic thickness (cm)	6.6 ± 1.4	9.1 ± 2.6
Etiology	Congestive splenomegaly (11) - Liver cirrhosis (10) - Chronic hepatitis (1) Immune-related splenomegaly (8) - Infectious mononucleosis (2) - Syphilis (1) - Familial mediterranean fever (1) - Crohn’s disease (1) - Chronic kidney transplant rejection (2) - Acute urticaria with angioedema (1)	Lymphoma (9) - Non-Hodgkin lymphoma (7) - Hodgkin’s disease (2) Myeloproliferative neoplasm (9) - Essential thrombocytosis (4) - Polycythemia vera (3) - Primary myelofibrosis (2) Leukemia (2) - CLL (1) - AML (1) Multiple myeloma (1)

BMI, body mass index; CLL, chronic lymphocytic leukemia; AML, acute myeloid leukemia.

**Table 2:** Comparison of MAV between different groups in 40 study patients.

Group/Subgroup	(*n*)	MAV (m/s)			Average Depth of Measurements (mean ± SD in cm)
		Mean ± SD	Minimum	Maximum	
Benign etiologies	19	2.23 ± 0.76	1.83	4.30	4.03 ± 0.65
Congestive splenomegaly	11	3.52 ± 0.47	2.65	4.30	4.30 ± 0.59
Immune-mediated splenomegaly	8	2.84 ± 0.92	1.83	4.29	3.65 ± 0.56
Malignant infiltrative splenomegaly	21	3.25 ± 0.68	1.65	4.66	3.84 ± 0.79

MAV, mean ARFI (acoustic radiation force impulse) velocity; SD, standard deviation.

**Table 3:** Summary of previous studies on the use of elastography to differentiate benign and malignant causes of splenomegaly in adults. kPa, kilopascal; 2D SWE, two-dimensional shear wave elastography; ARFI, acoustic radiation force impulse; SP, splenomegaly patients; HS, healthy subjects; CS, congestive splenomegaly; IRS, immune-related splenomegaly; MIS, malignant infiltrative splenomegaly.

Study	Number of Subjects		Elastographic Technique	N of Shots	Unit	Splenic Stiffness				*p*-Value
	SP	HS				CS (*n*)	IRS (*n*)	HS (*n*)	MIS (*n*)	
Webb et al., (2015) [15]	20	8	FibroScan (Echosens)	10	median in kPa	58.5 (11)	-	13.5 (8)	41.3 (9)	>0.05
			2D SWE (SuperSonic)	4		40.5 (11)	-	18.1 (8)	32.9 (9)	>0.05
Batur et al., (2019) [14]	56	17	ARFI (Siemens)	9	median in m/s	3.27 (19)	2.45 (16)	2.15 (17)	2.96 (21)	=0.001
Yalçın et al., (2020) [16]	61	20	ARFI (Siemens)	9	median in m/s	3.85 (21)	2.66 (17)	2.22 (20)	3.42 (23)	<0.001

## Data Availability

Data are available upon reasonable request from the corresponding author.
